# Long-Term Exposure to Traffic-Related Air Pollution and the Risk of Coronary Heart Disease Hospitalization and Mortality

**DOI:** 10.1289/ehp.1002511

**Published:** 2010-11-16

**Authors:** Wen Qi Gan, Mieke Koehoorn, Hugh W. Davies, Paul A. Demers, Lillian Tamburic, Michael Brauer

**Affiliations:** 1 School of Environmental Health; 2 School of Population and Public Health and; 3 Centre for Health Services and Policy Research, University of British Columbia, Vancouver, British Columbia, Canada

**Keywords:** air pollution, cohort studies, coronary heart disease, particulate matter, vehicle emissions

## Abstract

**Background:**

Epidemiologic studies have demonstrated that exposure to road traffic is associated with adverse cardiovascular outcomes.

**Objectives:**

We aimed to identify specific traffic-related air pollutants that are associated with the risk of coronary heart disease (CHD) morbidity and mortality to support evidence-based environmental policy making.

**Methods:**

This population-based cohort study included a 5-year exposure period and a 4-year follow-up period. All residents 45–85 years of age who resided in Metropolitan Vancouver during the exposure period and without known CHD at baseline were included in this study (*n* = 452,735). Individual exposures to traffic-related air pollutants including black carbon, fine particles [aerodynamic diameter ≤ 2.5 μm (PM_2.5_)], nitrogen dioxide (NO_2_), and nitric oxide were estimated at residences of the subjects using land-use regression models and integrating changes in residences during the exposure period. CHD hospitalizations and deaths during the follow-up period were identified from provincial hospitalization and death registration records.

**Results:**

An interquartile range elevation in the average concentration of black carbon (0.94 × 10^−5^/m filter absorbance, equivalent to approximately 0.8 μg/m^3^ elemental carbon) was associated with a 3% increase in CHD hospitalization (95% confidence interval, 1–5%) and a 6% increase in CHD mortality (3–9%) after adjusting for age, sex, preexisting comorbidity, neighborhood socioeconomic status, and copollutants (PM_2.5_ and NO_2_). There were clear linear exposure–response relationships between black carbon and coronary events.

**Conclusions:**

Long-term exposure to traffic-related fine particulate air pollution, indicated by black carbon, may partly explain the observed associations between exposure to road traffic and adverse cardiovascular outcomes.

A number of epidemiologic studies have demonstrated that long-term exposure to road traffic as indicated by residential proximity to major roadways or residential traffic intensity is associated with adverse cardiovascular outcomes including coronary artery atherosclerosis (Hoffmann et al. 2007), deep vein thrombosis (Baccarelli et al. 2009), fatal and nonfatal coronary events (Kan et al. 2008; Tonne et al. 2007), and cardiopulmonary mortality (Gehring et al. 2006; Hoek et al. 2002). In a previous analysis of this population-based cohort, Gan et al. (2010) observed that living close to road traffic was associated with an increased risk of coronary heart disease (CHD) mortality and that change in residential proximity to road traffic was associated with an altered risk of CHD mortality: moving close to traffic was associated with an increased risk, whereas moving away from traffic was associated with a decreased risk. In addition to exposure to traffic noise (Selander et al. 2009), residential proximity to road traffic may reflect exposure to multiple traffic-related air pollutants (Brauer et al. 2003; Künzli et al. 2000; Zhu et al. 2002). Identifying traffic-related air pollutants responsible for adverse cardiovascular outcomes is important for evidence-based environmental policy making and cost-effective air pollution intervention.

Metropolitan Vancouver, located on the west coast of Canada, has relatively low levels of air pollution compared with other metropolitan areas. For example, in this region, the annual average concentration of fine particles [aerodynamic diameter ≤ 2.5 μm (PM_2.5_)] is 5 μg/m^3^ (Brauer et al. 2008), in contrast to 8.7 μg/m^3^ in Toronto, Canada (Jerrett et al. 2009), 14.0 μg/m^3^ in metropolitan areas of the United States (Pope et al. 2004), 28.3 μg/m^3^ in the Netherlands (Beelen et al. 2008), and 22.8 μg/m^3^ in the Ruhr area, Germany (Hoffmann et al. 2007). As in most urban areas, motor vehicles are recognized as a major contributor to ambient air pollution and are responsible for much of the spatial variability in pollutant concentrations in this region (Henderson et al. 2007).

Based on our previous analyses (Gan et al. 2010), we conducted a large population-based cohort study to identify specific traffic-related air pollutants that might be responsible for the observed association between exposure to road traffic and the risk of CHD mortality. We also examined the relationships between traffic-related air pollutants and the risk of CHD hospitalization.

## Materials And Methods

### Study design

This population-based cohort study included two periods: a 5-year exposure period (January 1994–December 1998) and a 4-year follow-up period (January 1999–December 2002) for which mortality data were available. Average concentrations of traffic-related air pollutants were estimated at residences of the subjects using land-use regression (LUR) models and integrating changes in residences during the exposure period. Hospitalization and mortality information during the follow-up period was retrieved from provincial hospitalization records and death registration records, respectively. This study was approved by the Institutional Review Board of The University of British Columbia (Behavioural Research Ethics Board certificate H08-00185).

### Population

As described previously, we used linked administrative databases from the universal health insurance system of British Columbia to assemble a population-based cohort (Gan et al. 2010). All Metropolitan Vancouver residents who met the following criteria at baseline (January 1999) were included in the cohort: registered with the provincial health insurance plan, which provides universal coverage to nearly all residents in the study region; resided in the study region during the 5-year exposure period; 45–85 years of age; and no previous diagnosis of CHD.

### Air pollution exposure assessment

We used a high-resolution LUR model combined with residential histories to estimate individual exposure to traffic-related air pollutants including black carbon, PM_2.5_, nitrogen dioxide (NO_2_), and nitric oxide (NO) during the 5-year exposure period. This method has been described in detail elsewhere (Brauer et al. 2008; Henderson et al. 2007; Larson et al. 2009). Briefly, NO and NO_2_ concentrations were measured using Ogawa passive samplers (Ogawa USA, Pompano Beach, FL, USA) at 116 sites. PM_2.5_ concentrations were measured using Harvard Impactors (Air Diagnostics and Engineering, Harrison, ME, USA) at a subset of 25 locations. Light-absorbing carbon (black carbon) concentrations were measured using a particle soot absorption photometer (Radiance Research, Seattle, WA, USA) in a mobile monitoring campaign at a subset of 39 sites during the summer season (Larson et al. 2009). In the study region, the concentrations of black carbon based on the particle light absorption coefficient are highly correlated with the concentrations of elemental carbon measured by traditional thermal/optical reflectance (*R*^2^ = 0.7–0.8); 10^−5^/m black carbon is approximately equivalent to 0.8 μg/m^3^ elemental carbon (Rich 2002). Based on these measurements and after adjusting for temporal variation, we calculated annual average concentrations of these pollutants for each site.

Meanwhile, a total of 55 variables were generated in a geographic information system (GIS) (ArcGIS; ESRI, Redlands, CA, USA) to describe the land use characteristics of each site. Measured air pollutant concentrations and the most predictive land use characteristics were modeled using multiple linear regression techniques. As described previously (Brauer et al. 2008), we used the coefficient of determination (*R^2^*) and estimated mean error from leave-one-out cross validation analysis to evaluate the performance of these models. Overall, the performance was similar to those of previous studies (Hoek et al. 2008). For NO [*R*^2^ = 0.62, mean error (± SD) = 2.02 ± 15.5 μg/m^3^], the model included the length of highways within a 100-m and a 1,000-m radius, the length of major roads within a 100-m radius, the population density within a 2,500-m radius, around each sampling site, and the elevation of each site. For NO_2_ (*R*^2^ = 0.56, mean error = 0 ± 5.2 μg/m^3^), the model included all variables in the NO model and also the area of commercial land within a 750-m radius. For PM_2.5_ (*R*^2^ = 0.52, mean error = 0 ± 1.50 μg/m^3^), the model included the areas of commercial and industrial land within a 300-m radius, the area of residential land within a 750-m radius, and the elevation. For black carbon (*R*^2^ = 0.56, mean error = 0 ± 0.23 × 10^−5^/m), the model included the length of major roads within a 100-m radius, distance to the nearest highway, and the area of industrial land within a 750-m radius. Overall, the performance (SD of mean error/sample mean) of the models for NO (10%), NO_2_ (18%), and black carbon (14%) was better than that for PM_2.5_ (36%).

Based on the LUR models, we generated a predicted spatial surface for annual average concentrations for each pollutant in a GIS with a resolution of 10 m. We then applied month–year adjustment factors derived from regulatory monitoring data to estimate monthly concentrations. The monthly air pollution data were assigned to subjects through their six-digit residential postal codes (area centroids). In urban areas of Metropolitan Vancouver, a six-digit postal code represents one side of a city block, but may represent a larger area in less densely populated regions. After integrating changes in residences, we calculated average concentrations of black carbon, PM_2.5_, NO_2_, and NO during the 5-year exposure period for each study subject.

Because the air pollution exposure assessment did not cover the whole study region, air pollution data were not available for a small proportion of study subjects. These subjects were thus excluded from the analyses. Meanwhile, because of changes in residences, some subjects had partially missing air pollution data; those with missing data in more than a total of any 15 months or in more than 3 consecutive months during the 5-year exposure period were also excluded from the analyses.

### Case definitions

The outcomes of this study included CHD hospitalizations and CHD deaths that occurred during the 4-year follow-up period.

A CHD hospitalization case is a record of hospitalization with the following *International Statistical Classification of Diseases, 9th Revision* codes, ICD-9, 410–414 and 429.2 [World Health Organization (WHO) 1977] or *10th Revision* (ICD-10), I20–I25 (WHO 2007), as the principal diagnosis (the most responsible diagnosis) for a hospital admission in the provincial hospitalization database.

A CHD death is a death record with CHD as the cause of death in the provincial death registration database.

A broader definition was used to identify prior CHD cases. Subjects who had a hospitalization record with CHD as the principal or primary (the diagnosis that had a substantial influence on hospital length of stay) diagnosis before baseline (based on data from January 1991 to December 1998) were regarded as previously diagnosed CHD cases. These prior cases were excluded from the analysis to examine the association of incident CHD with traffic-related air pollution.

### Covariates

We included age, sex, preexisting comorbidity, and neighborhood socioeconomic status (SES) as covariates in the data analysis. We used the following ICD codes to identify preexisting comorbidity including diabetes (Pearson et al. 2002) (ICD-9, 250; ICD-10, E10–E14), chronic obstructive pulmonary disease (COPD) (Hole et al. 1996) (ICD-9, 490–492 and 496; ICD-10, J40–J44), and hypertensive heart disease (Pearson et al. 2002) (ICD-9, 401–404; ICD-10, I10–I13) that are independent risk factors for CHD. In addition, these chronic diseases and CHD share common behavioral risk factors such as cigarette smoking. Given a lack of individual data on behavioral risk factors in this study, we used the preexisting comorbidity as a proxy variable of common behavioral risk factors (Pope et al. 2009). To sufficiently control for the influence of the comorbidities and the common behavioral risk factors, all diagnoses in a hospitalization record (up to 16 diagnoses before 2001 and up to 25 diagnoses since 2001) were used to identify subjects with these comorbidities. One hospitalization record with the diagnosis of any of these diseases during January 1991–December 1998 was defined as the presence of comorbidity.

Neighborhood SES reflects neighborhood disadvantages and is a risk factor for CHD (Diez Roux et al. 2001; Sundquist et al. 2004). In addition, because individual SES data were not available in this study, we used neighborhood SES to approximate individual SES (Domínguez-Berjón et al. 2006; Krieger 1992). The neighborhood income quintiles from the 2001 Statistics Canada Census were assigned to study subjects using their residential postal codes. For the 2001 Census, a dissemination area with 400–700 persons was the smallest census geographic unit for which all census data were disseminated. Within a census metropolitan area, all dissemination areas were ranked by household size–adjusted average family income and divided into quintiles (Gan et al. 2010).

### Statistical analysis

The baseline characteristics between study subjects with different outcomes were compared using a chi-square test for categorical variables and *t* test for continuous variables. Correlations between these pollutants were examined using Spearman’s rank correlation.

The Cox proportional hazards regression model was used to determine the associations of each air pollutant with CHD hospitalization and mortality. CHD hospitalization and CHD death were regarded as independent events; for CHD hospitalization analysis, CHD deaths without a hospitalization record were treated as censored cases like those who died from other diseases; for CHD mortality analysis, CHD hospitalization cases without a death record were treated the same way as those without a CHD event. Person-years were calculated for study subjects from baseline to the date of the first CHD hospitalization, CHD death, or end of follow-up. For those who died from other diseases or those who moved out of the province, person-years were calculated from baseline to the date of death or the last known date in the province. We first calculated relative risks (RRs) of CHD events in response to an interquartile range (IQR) elevation in the average concentration of each pollutant using bivariable and multivariable models. In the multivariable analysis, we gradually adjusted for age, sex, preexisting comorbidity (diabetes, COPD, or hypertensive heart disease), neighborhood income quintiles, and copollutants. We further examined exposure–response relationships by dividing study subjects into quintiles based on the concentrations of each pollutant. RRs of CHD events were calculated for quintile 2 to quintile 5, using quintile 1 (lowest) as the reference category. Linear trend across quintile groups was examined by using quintiles of a pollutant as a continuous variable.

For those pollutants strongly associated with CHD hospitalization and mortality, we performed stratification analyses to examine effect modification by age, sex, preexisting comorbidity, and neighborhood SES. In this analysis, age was categorized into three groups (< 60, 60–69, ≥ 70 years) as used in previous studies (Miller et al. 2007; Pope et al. 2002). Neighborhood SES was categorized into two groups: low (neighborhood income quintile 1–3) and high (neighborhood income quintile 4–5).

All statistical analyses were performed using SAS 9.2 software (SAS Institute Inc., Cary, NC, USA).

## Results

At baseline, a total of 466,727 subjects who met the inclusion criteria were included in this study. Among these subjects, 13,992 (3.0%) with missing air pollution data were excluded, which left 452,735 subjects for the present analysis. During the 4-year follow-up period, 17,542 (3.9%) moved out of the province and 16,367 (3.6%) died from other diseases, leaving 418,826 (92.5%) subjects at the end of follow-up. Of these subjects, 45.9% were male; the average age (SD) was 58.9 (10.5) years (range, 45–83 years).

Although multiple ICD-9 and ICD-10 codes were used to identify CHD cases, acute myocardial infarction (ICD-9 code 410 and ICD-10 codes I21, I22) was the leading cause of hospitalization (41.2%) and death (56.8%). Compared with the subjects without CHD event, hospitalization cases and death cases were older and more likely to be male and have preexisting comorbidity and lower neighborhood SES, especially for death cases (Table 1).

Descriptive statistics and Spearman’s rank correlation coefficients for these pollutants are summarized in Table 2. Overall, except for the correlation between NO_2_ and NO, these pollutants were weakly correlated with each other.

### Traffic-related air pollution and CHD hospitalization

During the follow-up period, 10,312 subjects were hospitalized for CHD (hospitalization rate, 6.0 per 1,000 person-years). Exposure to black carbon was associated with CHD hospitalization. For an IQR elevation in black carbon concentration (0.94 × 10^−5^/m), CHD hospitalization increased 4% [95% confidence interval (CI), 3–6%]. Adjusting for age, sex, preexisting comorbidity, and neighborhood SES reduced the effect estimate, whereas additional adjustment for copollutants (PM_2.5_ and NO_2_) increased the effect estimate (Table 3). PM_2.5_ was similar to black carbon in the magnitude of association with CHD hospitalization; whereas NO_2_ and NO were inversely associated with CHD hospitalization in adjusted models (Table 3).

**Figure 1:**
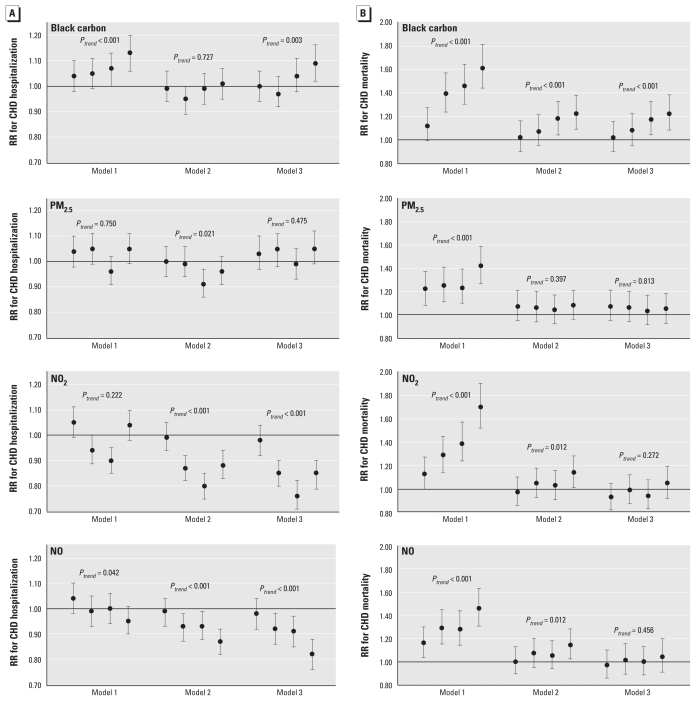
RRs and 95% CIs of CHD hospitalization (*A*) and mortality (*B*) for quintiles of black carbon, PM_2.5_, NO_2_ and NO. Quintile 1 (lowest) was the reference category. From left to right, each error bar represents RR and 95% CI of CHD hospitalization (*A*) or mortality (*B*) for quintiles 2–5, respectively, compared with quintile 1. *p_trend_* indicates linear trend across quintile groups. Model 1, bivariable analysis; model 2, adjusted for age, sex, preexisting comorbidity, and neighborhood SES; model 3, additionally adjusted for copollutants (PM_2.5_ and NO_2_ for black carbon, black carbon and NO_2_ for PM_2.5_, black carbon and PM_2.5_ for NO_2_ and NO).

CHD hospitalization gradually increased in response to quintiles of black carbon concentrations in bivariable and fully adjusted models, but not in the partially adjusted model (Figure 1A). In contrast, there was no linear exposure–response relationship between PM_2.5_ and CHD hospitalization and some evidence of inverse associations of NO_2_ and NO with CHD hospitalization (Figure 1A).

**Figure 2:**
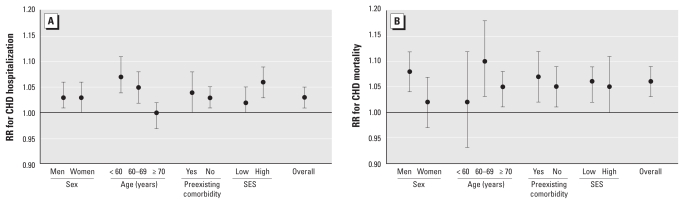
Adjusted RRs and 95% CIs for CHD hospitalization (*A*) and mortality (*B*) associated with an IQR elevation in black carbon concentration, stratified by each covariate and adjusted for all other covariates in the figure and copollutants PM_2.5_ and NO_2_.

Stratification analysis shows that CHD hospitalization in response to an IQR elevation in black carbon concentrations was higher for people < 70 years of age and for those living in the areas with higher neighborhood SES (Figure 2A).

### Traffic-related air pollution and CHD mortality

A total of 3,104 subjects died from CHD (mortality rate, 1.8 per 1,000 person-years) during the follow-up period. Exposure to black carbon was strongly associated with CHD mortality. For an IQR elevation in black carbon concentration (0.94 × 10^−5^/m), CHD mortality increased 14% (95% CI, 11–17%). Adjusting for age, sex, preexisting comorbidity, and neighborhood SES greatly reduced the effect estimate; additional adjustment for copollutants (PM_2.5_ and NO_2_) did not change the effect estimate (Table 3). NO_2_ and NO (but not PM_2.5_) had a similar magnitude of association with CHD mortality.

We also observed a strong exposure–response relationship between exposure to black carbon and CHD mortality in bivariable and multivariable models (Figure 1B). For NO_2_ and NO, an exposure–response relationship was present in the bivariable models and in the multivariable models including age, sex, preexisting comorbidity, and neighborhood SES, but not after further adjustment for black carbon and PM_2.5_ (Figure 1B). For PM_2.5_, a linear trend was evident in the bivariable model but not in any of the adjusted models (Figure 1B).

Stratification analysis shows that CHD mortality associated with an IQR elevation in black carbon concentration was higher for men and for those 60–69 years of age, although there was considerable overlap in the risk estimates (Figure 2B).

During the 4-year follow-up period, there was no evident change in traffic-related air pollution such as PM_2.5_ and NO_2_ in this study region (Greater Vancouver Regional District 2003). Our exposure assessment accounted for changes in residences during the exposure period. Futher, a sensitivity analysis showed that the effect estimates remain unchanged after excluding those who changed their residences during the 4-year follow-up period.

## Discussion

This large population-based cohort study demonstrated that long-term exposure to higher concentrations of black carbon was associated with increased risks of CHD hospitalization and mortality in an exposure–response fashion. The observed association with CHD mortality was particularly strong.

Black carbon results mainly from incomplete combustion of diesel fuels and is a surrogate for diesel exhaust particles (Schauer 2003). It may also be emitted from other sources such as gasoline-powered vehicles and wood combustion (Schauer 2003). Metropolitan Vancouver is a highly urbanized region; road traffic is the predominant source of black carbon and determines much of the spatial variability in the concentrations, especially during the summer season. In general, black carbon can be regarded as an indicator of the traffic-related component of fine particulate air pollution (Gold et al. 2005; Schwartz et al. 2005).

A recent case–control study used measured black carbon and NO_2_ levels to estimate traffic particle levels and found that an IQR (0.2 × 10^−5^/m) elevation in modeled traffic particle concentration was associated with a 10% (95% CI, 4–16%) increase in acute myocardial infarction (Tonne et al. 2009). In a 9-year Dutch cohort study, a 10-μg/m^3^ increase in annual average concentration of black smoke was associated with a nonsignificant 4% increase in cardiovascular mortality (Beelen et al. 2008). A recent time-series study of 12 million Medicare enrollees in 119 U.S. urban communities found that an IQR (0.4 μg/m^3^) elevation in daily elemental carbon concentration was associated with a 0.8% (95% CI, 0.3–1.3%) increase in same-day cardiovascular hospitalizations. Elemental carbon was the only component of PM_2.5_ associated with cardiovascular hospitalizations (Peng et al. 2009). Similarly, in a time-series study, Laden et al. (2000) observed that traffic-related fine particles were more strongly associated with CHD mortality than with respiratory mortality, whereas coal-derived fine particles were more strongly associated with respiratory mortality than with CHD mortality. The findings of our study are consistent with those from previous studies, demonstrating that black carbon, as an indicator of traffic-related fine particulate air pollution, may be partly responsible for the observed associations between exposure to road traffic and adverse cardiovascular outcomes.

There is also strong evidence linking black carbon to various subclinical pathophysiological responses. Controlled exposure studies in healthy human volunteers demonstrated that short-term exposure to diesel exhaust can cause acute artery vasoconstriction (Peretz et al. 2008), vascular endothelial dysfunction (Mills et al. 2005; Törnqvist et al. 2007), and marked pulmonary and systemic inflammation (Nightingale et al. 2000; Salvi et al. 1999; Törnqvist et al. 2007). Further, exposure to ambient black carbon or elemental carbon in fine particles has been associated with airway (Jansen et al. 2005) and systemic inflammation (Delfino et al. 2008), platelet activation (Delfino et al. 2008), plasma homocysteine (Park et al. 2008), heart rate variability (Schwartz et al. 2005), cardiac arrhythmia (Dockery et al. 2005), and myocardial ischemia (Chuang et al. 2008; Gold et al. 2005; Mills et al. 2007). These findings suggest multiple biological mechanisms for the associations between black carbon and coronary events.

We did not find evidence of a linear exposure–response relationship between PM_2.5_ and CHD hospitalization or mortality, as reported in some previous studies (Miller et al. 2007; Pope et al. 2002). This finding was, however, consistent with the results of several other studies (Beelen et al. 2008; Hoffmann et al. 2007; Jerrett et al. 2009). As mentioned before, in this study region, PM_2.5_ levels were substantially lower compared with those of other metropolitan areas. In addition, road traffic was just one of numerous sources for ambient PM_2.5_. Therefore, the spatial distribution of PM_2.5_ is relatively more homogeneous. The null exposure–response relationship between PM_2.5_ and CHD probably reflects the inability of our exposure assessment method to differentiate spatial variability of PM_2.5_ in this intra-urban study.

Some studies have reported associations between long-term residential exposure to NO_2_ (Rosenlund et al. 2008, 2009) or NO_x_ (Nafstad et al. 2004) and CHD mortality. In these studies, NO_2_/NO_x_ was used as a surrogate for within-city traffic-related air pollution. In our study, we also observed a linear exposure–response relationship between NO_2_ or NO and CHD mortality. However, this relationship was mostly attenuated after adjustment for black carbon, suggesting that black carbon played a more important role than NO_2_ and NO in association with CHD mortality in this study region.

This study has several strengths that support the validity of the findings. First, this large population-based cohort study included 452,735 subjects without known CHD at baseline. The large sample size and statistical power enabled this study to detect small adverse coronary effects with relatively higher precision. Second, this study used two different coronary outcomes, hospitalization and mortality (from different data sources), to evaluate the adverse effects of these pollutants. The associations between black carbon and these two outcomes were consistent. Third, we collected detailed residential history information. Average concentrations of air pollutants were calculated for each subject after integrating changes in residences during the 5-year exposure period. As previously demonstrated (Gan et al. 2010), this method can effectively reduce exposure misclassification from residence relocation. Fourth, we used LUR models with high spatial resolution for exposure assessment. This approach facilitates spatial variability of pollutant concentrations and provides increased exposure contrasts and statistical power.

This study also has some limitations. First, the cohort was constructed using provincial health insurance registry and linked administrative health databases. As previously discussed (Gan et al. 2010), some important information about individual cardiovascular risk factors such as cigarette smoking was not available and thus could not be controlled in data analysis. We adjusted for age, sex, preexisting comorbidity (diabetes, COPD, hypertensive heart disease), and neighborhood SES. Because these comorbidities and CHD share common behavioral risk factors, adjusting for these comorbidities to some extent was able to reduce the influence of some uncontrolled risk factors and these comorbidities themselves on the effect estimates (Pope et al. 2009). On the other hand, because diabetes, COPD, and hypertensive heart disease might serve as intermediate variables for the association between traffic-related air pollution and coronary events, adjusting for these comorbidities might lead to underestimations of the true adverse effects (Schisterman et al. 2009).

Second, cigarette smoking is the single most important risk factor for CHD and was not measured in this study (Ockene and Miller 1997). However, previous studies have shown that cigarette smoking did not substantially affect the associations between fine particulate air pollution and adverse cardiovascular outcomes such as coronary atherosclerosis (Hoffmann et al. 2007), carotid intima-media thickness (Künzli et al. 2005), and CHD mortality (Pope et al. 2004). Based on these previous findings, we believe that the observed associations in this study are unlikely to be due to the confounding effects of cigarette smoking.

Third, low individual SES is a risk factor for CHD (Kaplan and Keil 1993) and may be also related to exposure to traffic-related air pollution (Gunier et al. 2003). Individual SES is thus a possible confounder for the observed association. As mentioned before, because individual SES was not available in this study, we used neighborhood income quintiles to approximately estimate individual SES. There is some evidence that this approach is valid for control of individual SES (Domínguez-Berjón et al. 2006; Krieger 1992); however, this approach was unlikely to control all confounding effects related to individual SES (Geronimus and Bound 1998).

Fourth, like those used in previous studies, the exposure assessment in this study can only approximately reflect the exposure levels at subjects’ residences (postal code centroids). Many factors such as air infiltration, individual mobility, and outdoor activity might substantially affect actual individual exposure to traffic-related air pollution. This exposure assessment method did not take into account these individual factors and thus cannot precisely reflect actual individual exposure levels. Nevertheless, these factors are most likely to cause nondifferential exposure misclassification, leading to underestimations of the true adverse coronary effects (Van Roosbroeck et al. 2008).

Fifth, exposure to traffic-related air pollution may be associated with exposure to traffic noise (Davies et al. 2009). Some evidence has indicated that exposure to traffic noise is associated with CHD events (Selander et al. 2009). In the present study, traffic noise might also play a role in the association between black carbon and CHD events.

Sixth, because of privacy protection, we were unable to contact CHD cases or access their original medical records. As a result, we were unable to evaluate the accuracy of CHD diagnosis recorded in the provincial hospitalization database and death registration database. There were up to 16 diagnoses (1991–2000) or up to 25 diagnoses (2001–2002) in each hospitalization record. To reduce the possibility of misdiagnosis, we used only the principal diagnosis (the most responsible diagnosis for a hospital admission) to identify hospitalization cases. This stringent definition for hospitalization case might improve the accuracy of the CHD classification; however, we might inevitably lose some hospitalization cases for which CHD was not the principal diagnosis and thereby underestimate the true adverse effects.

Finally, although air pollution exposures were estimated based on residential postal codes, because of privacy protection, residential postal codes were eliminated from data files after data linkage. Therefore, we were unable to access residential postal codes of the subjects and cannot adjust for spatial clustering of the air pollution data, which might lead to underestimations of the standard errors in Cox regression models.

## Conclusions

This large, population-based cohort study demonstrated that long-term exposure to higher concentrations of black carbon was associated with an increased risk of CHD hospitalization and mortality in an exposure–response fashion. These findings suggest that traffic-related fine particulate air pollution, indicated by black carbon, may be partly responsible for the observed associations between exposure to road traffic and adverse cardiovascular outcomes.

## Figures and Tables

**Table 1:** Baseline characteristics of study subjects (%).

Characteristic	Subjects without CHD event (*n* = 406,232)	Hospitalization cases* (*n* = 10,312)	Mortality cases* (*n* = 3,104)
Men	45.3	66.4	61.5
Age (years)^a^	58.7 ± 10.4	65.4 ± 10.1	72.5 ± 8.9
Comorbidity			
Diabetes	1.8	7.9	13.3
COPD	1.0	2.8	9.8
Hypertensive heart disease	3.6	10.8	19.3
Any of the above	5.5	17.2	31.2
Income quintiles^b^			
1	17.9	19.8	26.2
2	18.9	19.5	21.6
3	19.5	19.4	18.3
4	20.7	20.7	18.1
5	23.1	20.5	15.8

a Data are presented as mean ± SD.

b Quintile 1 represents the lowest neighborhood income and quintile 5 the highest income.

* *p* < 0.05 for all comparisons with subjects without CHD event.

**Table 2:** Average concentrations of traffic-related air pollutants during the 5-year exposure period and Spearman correlation coefficients.

					Spearman correlation coefficient*			
Pollutant	Mean ± SD	Median	IQR	Range	BC	PM_2.5_	NO_2_	NO
BC (10^−5^/m)	1.49 ± 1.10^a^	1.02	0.94	0–4.98	1.00	–	–	–
PM_2.5_ (μg/m^3^)	4.08 ± 1.63	4.03	1.58	0–10.24	0.13	1.00	–	–
NO_2_ (μg/m^3^)	32.1 ± 8.0	30.6	8.4	15.3–57.7	0.39	0.47	1.00	–
NO (μg/m^3^)	32.0 ± 11.9	29.3	13.2	8.8–126.0	0.42	0.43	0.67	1.00

BC, black carbon.

a Equivalent to approximately 1.19 ± 0.88 μg/m^3^ elemental carbon (10^−5^/m black carbon ≈ 0.8 μg/m^3^ elemental carbon).

* *p* < 0.001 for each correlation coefficient.

**Table 3:** RRs (95% CIs) of CHD hospitalization and mortality for an IQR elevation in average concentrations of traffic-related air pollutants.

Model	BC (0.94 × 10^−5^/m)^a^	PM_2.5_ (1.58 μg/m^3^)^a^	NO_2_ (8.4 μg/m^3^)^a^	NO (13.2 μg/m^3^)^a^
Hospitalization				
Model 1: unadjusted single pollutant	1.04 (1.03–1.06)	1.03 (1.01–1.05)	1.02 (1.00–1.04)	0.99 (0.97–1.02)
Model 2: + sex, age, comorbidity, SES	1.01 (1.00–1.03)	1.00 (0.98–1.02)	0.97 (0.95–0.99)	0.96 (0.94–0.98)
Model 3: + two other pollutants^b^	1.03 (1.01–1.05)	1.02 (1.00–1.05)	0.96 (0.94–0.98)	0.95 (0.92–0.97)
Mortality				
Model 1: unadjusted single pollutant	1.14 (1.11–1.17)	1.13 (1.09–1.16)	1.19 (1.15–1.23)	1.13 (1.09–1.17)
Model 2: + sex, age, comorbidity, SES	1.06 (1.03–1.09)	1.01 (0.98–1.05)	1.04 (1.01–1.08)	1.06 (1.02–1.10)
Model 3: + two other pollutants^b^	1.06 (1.03–1.09)	1.00 (0.96–1.03)	1.03 (0.99–1.07)	1.03 (0.99–1.08)

+ additionally adjusted for covariates.

a IQR.

b Additionally adjusted for PM_2.5_ and NO_2_ for black carbon, black carbon and NO_2_ for PM_2.5_, black carbon and PM_2.5_ for NO_2_ and NO.
